# Supplementary figures and images for: Visualizing Microbial Community Dynamics via a Controllable Soil Environment
Source: mSystems. 2020 Feb 11;5(1):e00645-19. doi: 10.1128/mSystems.00645-19 (PMC7018529; doi:10.1128/mSystems.00645-19)

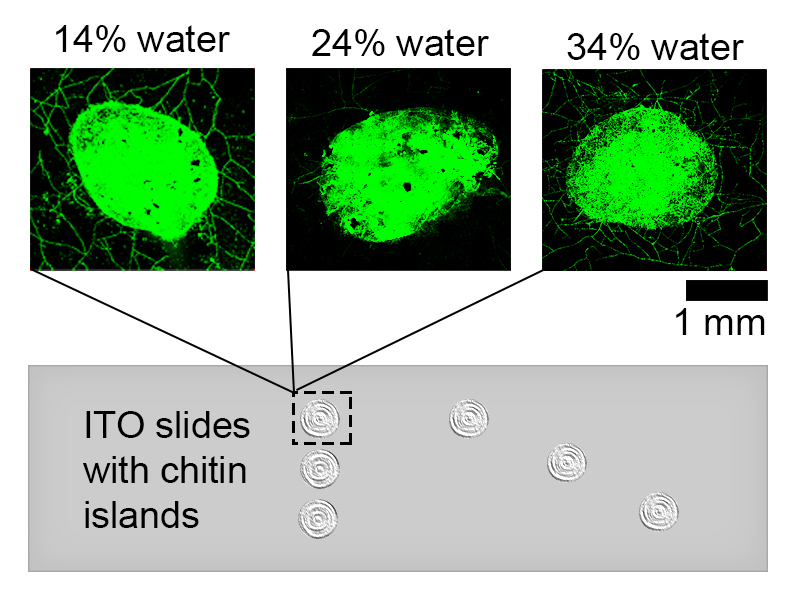

Supplement: FIG S1 [file mSystems.00645-19-sf001.jpg]

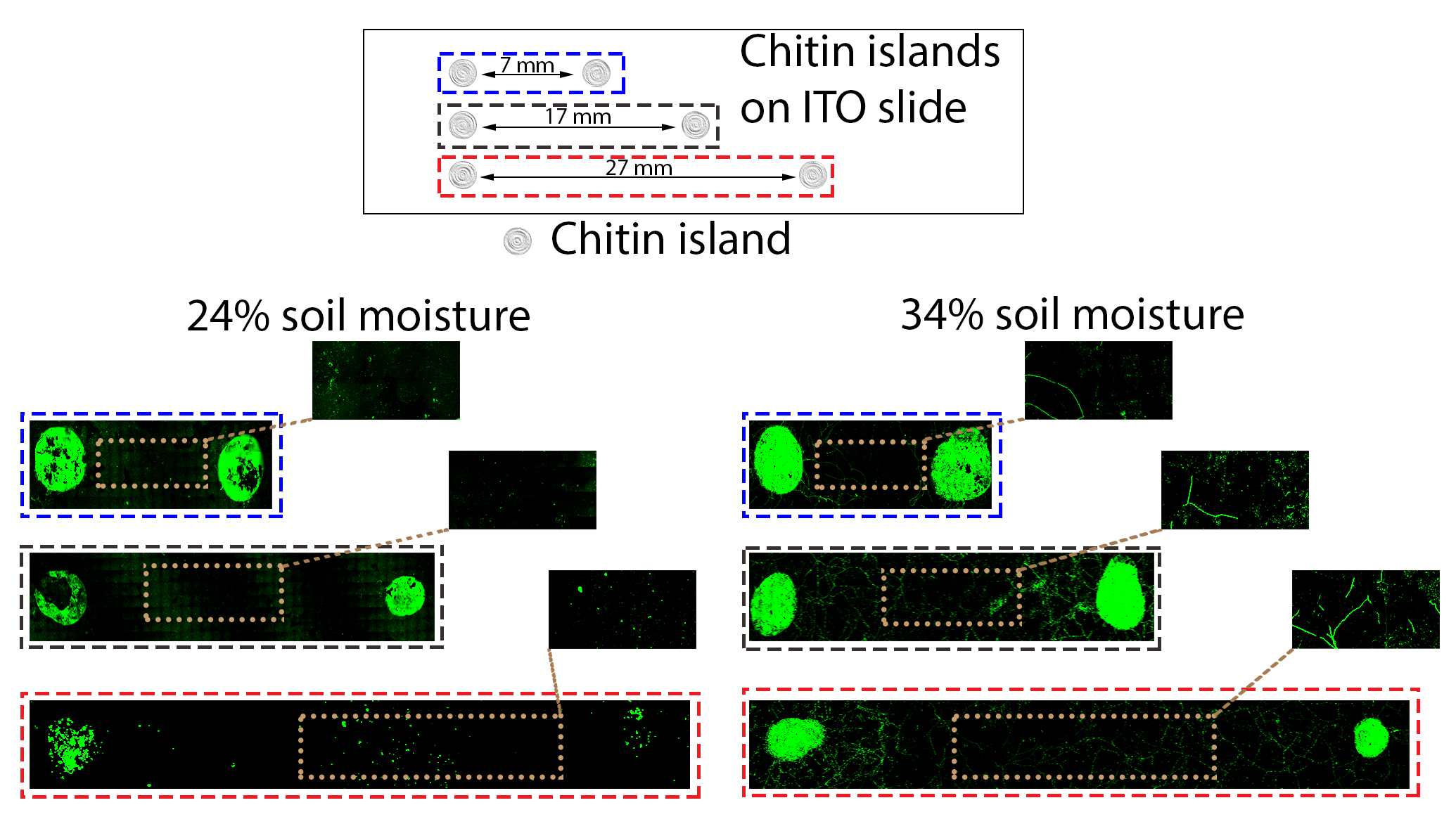

Supplement: FIG S2 [file mSystems.00645-19-sf002.jpg]

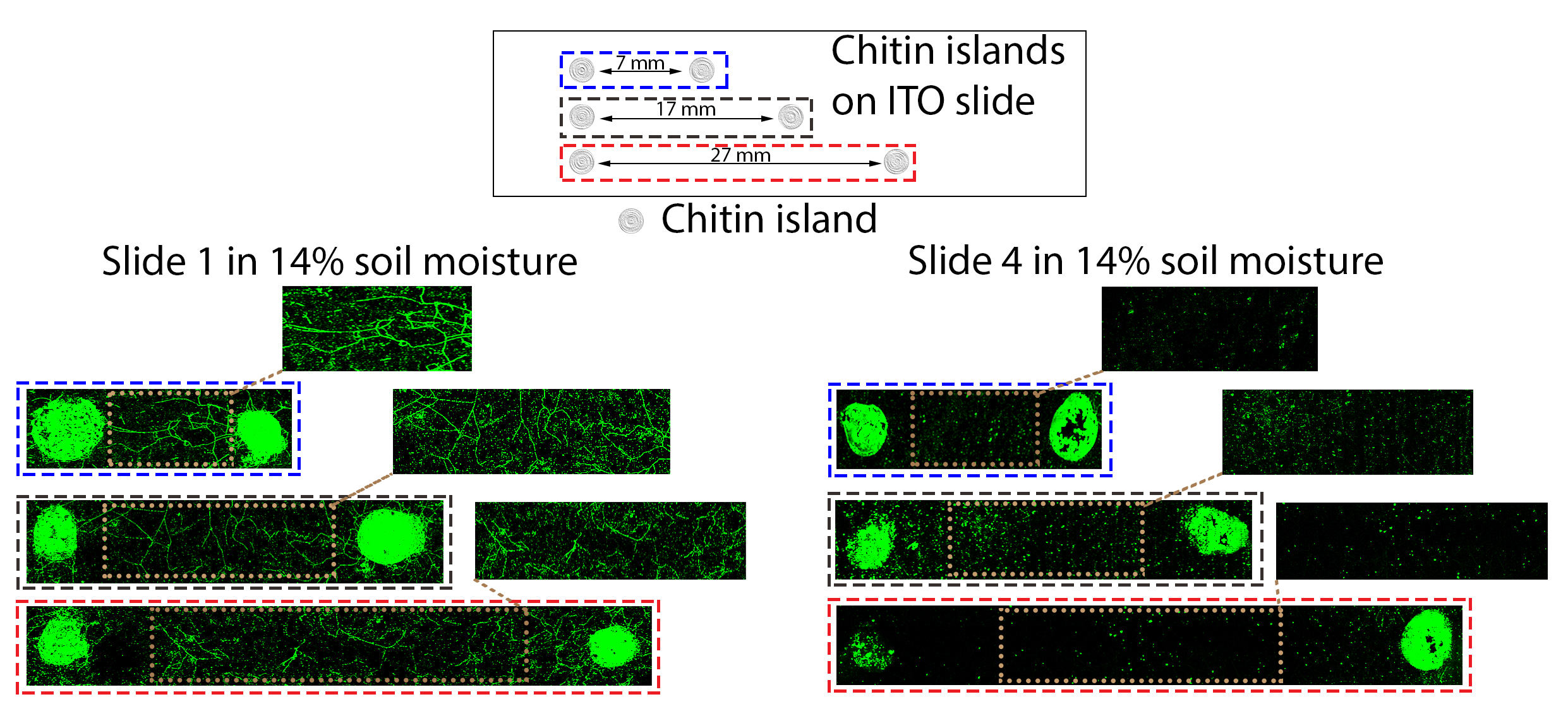

Supplement: FIG S3 [file mSystems.00645-19-sf003.jpg]

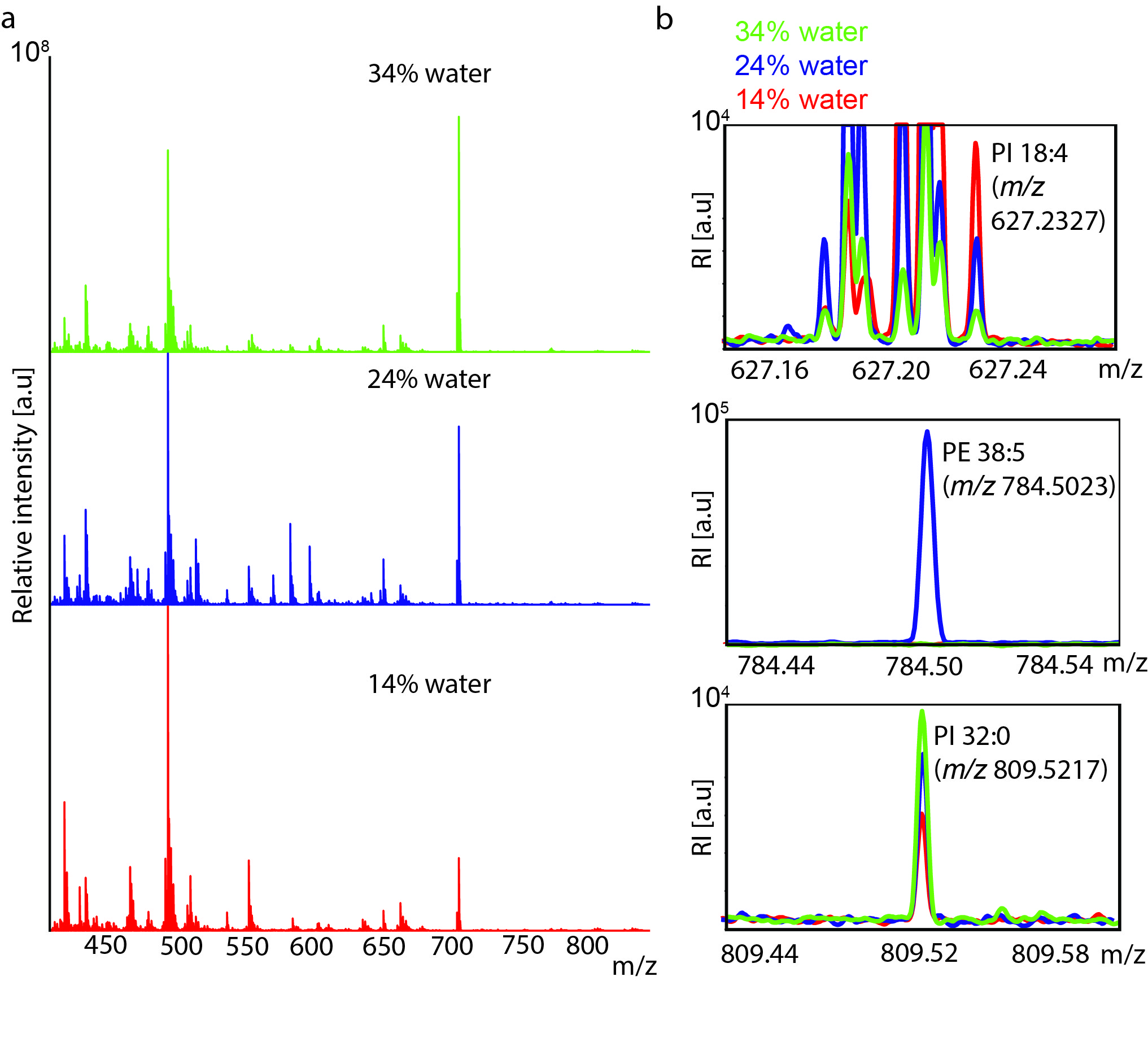

Supplement: FIG S4 [file mSystems.00645-19-sf004.jpg]

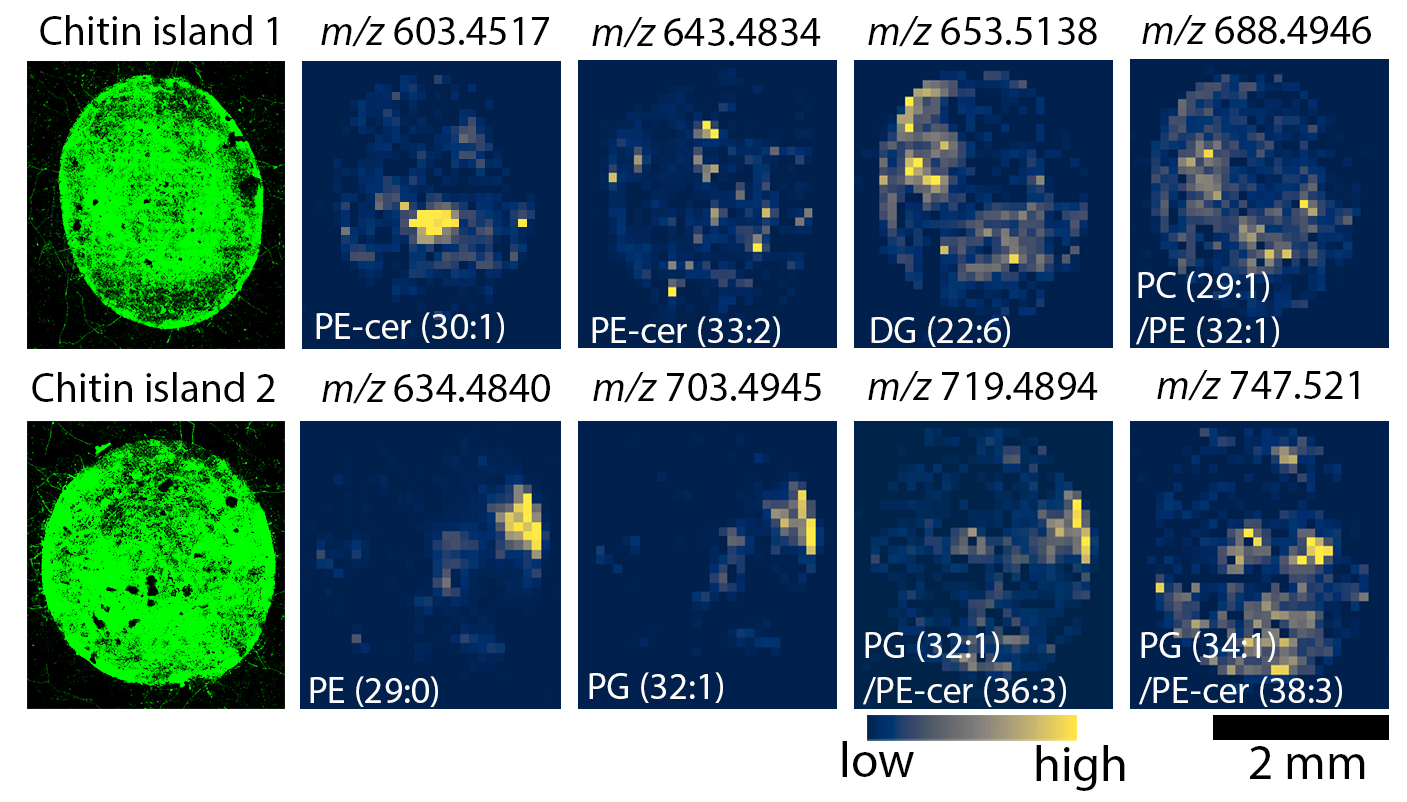

Supplement: FIG S5 [file mSystems.00645-19-sf005.jpg]

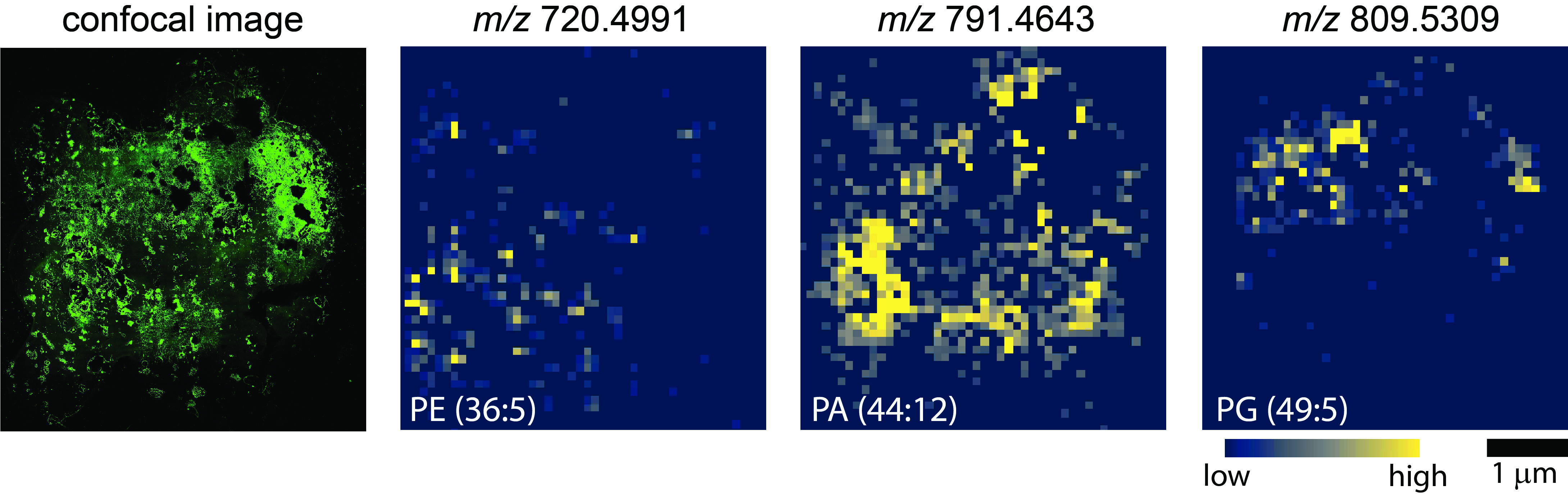

Supplement: FIG S6 [file mSystems.00645-19-sf006.jpg]

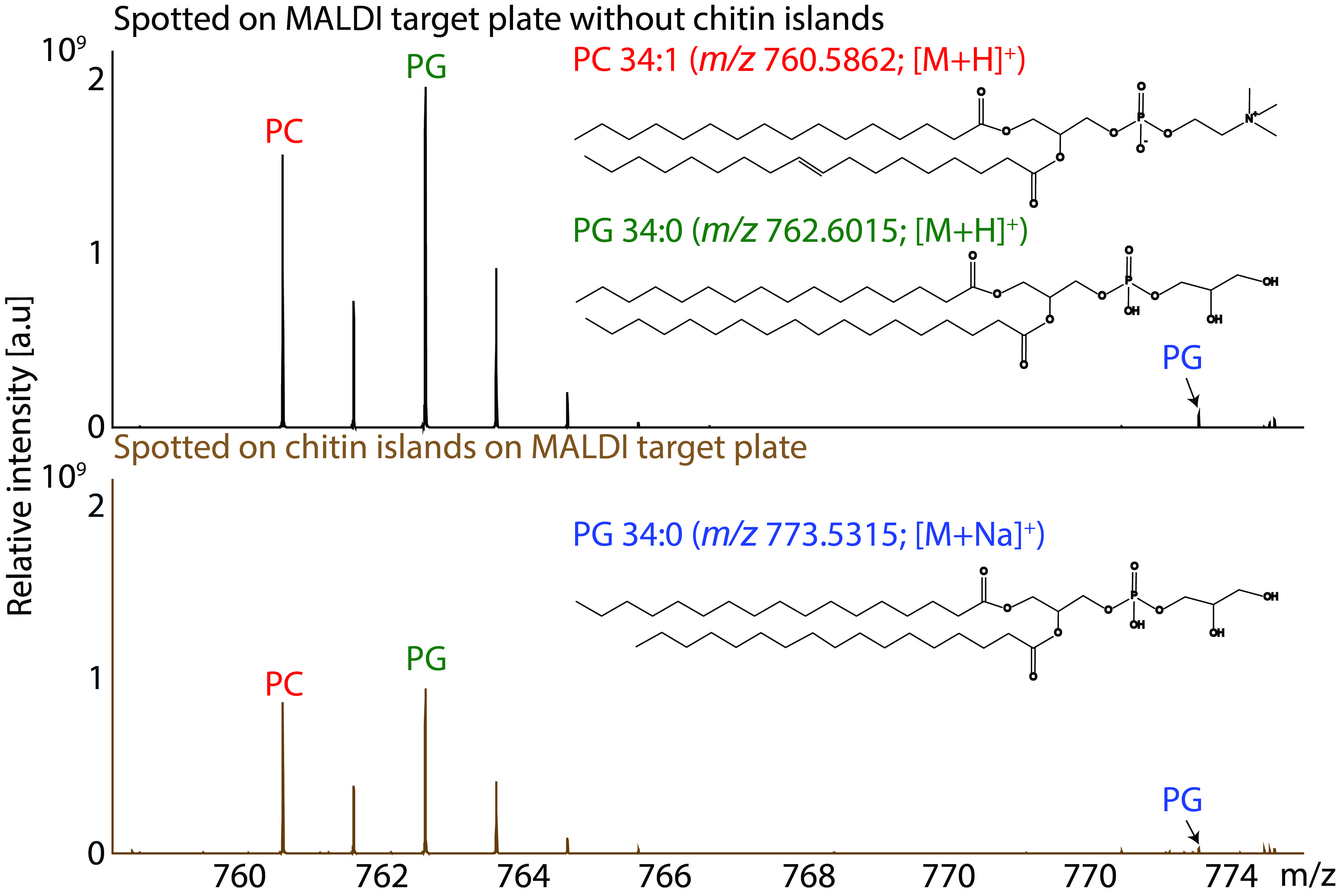

Supplement: FIG S7 [file mSystems.00645-19-sf007.jpg]
