# Supplementary material for: Visualizing Microbial Community Dynamics via a Controllable Soil Environment
Source: mSystems. 2020 Feb 11;5(1):e00645-19. doi: 10.1128/mSystems.00645-19 (PMC7018529; doi:10.1128/mSystems.00645-19)
Supplement: TABLE S1 [file mSystems.00645-19-st001.docx]

**Supplementary Information for:**

**Visualizing microbial community dynamics via a controllable soil environment**

Arunima Bhattacharjee^1^, Dusan Velickovic^1^, Thomas W. Wietsma^1^, Sheryl L. Bell^1^, Janet K. Jansson^2^, Kirsten S. Hofmockel^1,3^, Christopher R. Anderton^1*^

^1^Environmental Molecular Sciences Division; ^2^Biological Sciences Division, Earth and Biological Sciences Directorate, Pacific Northwest National Laboratory, Richland, WA; ^3^Department of Ecology, Evolution and Organismal Biology, Iowa State University, Ames IA, 50010

[*Christopher.Anderton@pnnl.gov](mailto:*Christopher.Anderton@pnnl.gov); 902 Battelle Boulevard, Richland, Washington 99354; 509-371-7970

**Table of contents:**

**Supplementary tables……………………………………………………………………………2**

Table 1**…………………………………………………………………………………………….2**

**Table S1.** The different lipids families, phosphatidylethanolamine (PE), phosphatidylglycerol (PG), PE-ceramides, phosphatidylinositol (PI), sphingomyelin (SM), and diglycerides (DG) detected using MALDI-MSI. The lipid ions, ppm error, their corresponding molecular formula and adduct were putatively annotated using the METLIN database (see MATERIALS AND METHODS). The last column denotes the dynamic range (relative ratio) of these lipids detected over different % humidity conditions in the SoilBoxes.

| **No.** | **Lipid** | ***m/z*** | **ppm error** | **Molecular formula** | **Adduct** | **Relative ratio among humidity conditions (14%/24%/34%)** |
| --- | --- | --- | --- | --- | --- | --- |
| 1 | PE-cer (30:1) | 603.4517 | 1 | C_32_H_65_N_2_O_6_P | [M-H]^-^ | 1/26/1.7 |
| 2 | PI (18:4) | 627.2327 | 2 | C_27_H_45_O_12_P | [M+Cl]^-^ | 5.8/3/1 |
| 3 | PE(29:0) | 634.4840 | 3 | C_34_H_70_NO_7_P | [M-H]^-^ | 1/14/3 |
| 4 | PE-cer (33:2) | 643.4834 | 2 | C_35_H_69_N_2_O_6_P | [M-H]^-^ | 3.7/40.6/1 |
| 5 | SM (30:1) | 645.4992 | 2 | C_35_H_71_N_2_O_6_P | [M-H]^-^ | 6/178/1 |
| 6 | PE-cer (33:1) | 645.4992 | 2 | C_35_H_71_N_2_O_6_P | [M-H]^-^ | 6/178/1 |
| 7 | DG (22:6) | 653.5138 | 1 | C_42_H_70_O_5_ | [M-H]^-^ | 1/84/18 |
| 8 | PE (32:1) | 672.5046 | 3 | C_37_H_72_NO_7_P | [M-H]^-^ | 1/7.6/2.3 |
| 9 | PC (29:1) | 688.4946 | 3 | C_37_H_72_NO_8_P | [M-H]^-^ | 5/11.4/1 |
| 10 | PE (32:1) | 688.4946 | 3 | C_37_H_72_NO_8_P | [M-H]^-^ | 5/11.4/1 |
| 11 | PG (32:1) | 703.4945 | 3 | C_38_H_73_O_9_P | [M-H]^-^ | 1/6.8/5.1 |
| 12 | PG (32:1) | 719.4894 | 3 | C_38_H_73_O_10_P | [M-H]^-^ | 1.5/3.6/1 |
| 13 | PE-cer (36:3) | 719.4894 | 0 | C_38_H_73_N_2_O_6_P | [M+Cl]^-^ | 1.5/3.6/1 |
| 14 | SM (33:2) | 721.5059 | 0 | C_38_H_75_N_2_O_6_P | [M+Cl]^-^ | 1.4/9.4/1 |
| 15 | PE-cer (36:2) | 721.5059 | 0 | C_38_H_75_N_2_O_6_P | [M+Cl]^-^ | 1.4/9.4/1 |
| 16 | PG (34:1) | 747.5210 | 3 | C_40_H_77_O_10_P | [M-H]^-^ | 1/12.5/3.3 |
| 17 | PE-cer (38:3) | 747.5210 | 0 | C_40_H_77_N_2_O_6_P | [M+Cl]^-^ | 1/12.5/3.3 |
| 18 | PE (38:5) | 784.5023 | 3 | C_43_H_76_NO_7_P | [M+Cl]^-^ | 0/265000/0 |
| 19 | PI (32:0) | 809.5217 | 3 | C_41_H_79_O_13_P | [M-H]^-^ | 1/1.6/2 |
