# Supplementary material for: Visualizing Microbial Community Dynamics via a Controllable Soil Environment
Source: mSystems. 2020 Feb 11;5(1):e00645-19. doi: 10.1128/mSystems.00645-19 (PMC7018529; doi:10.1128/mSystems.00645-19)
Supplement: FIG S8 [file mSystems.00645-19-sf008.pdf]

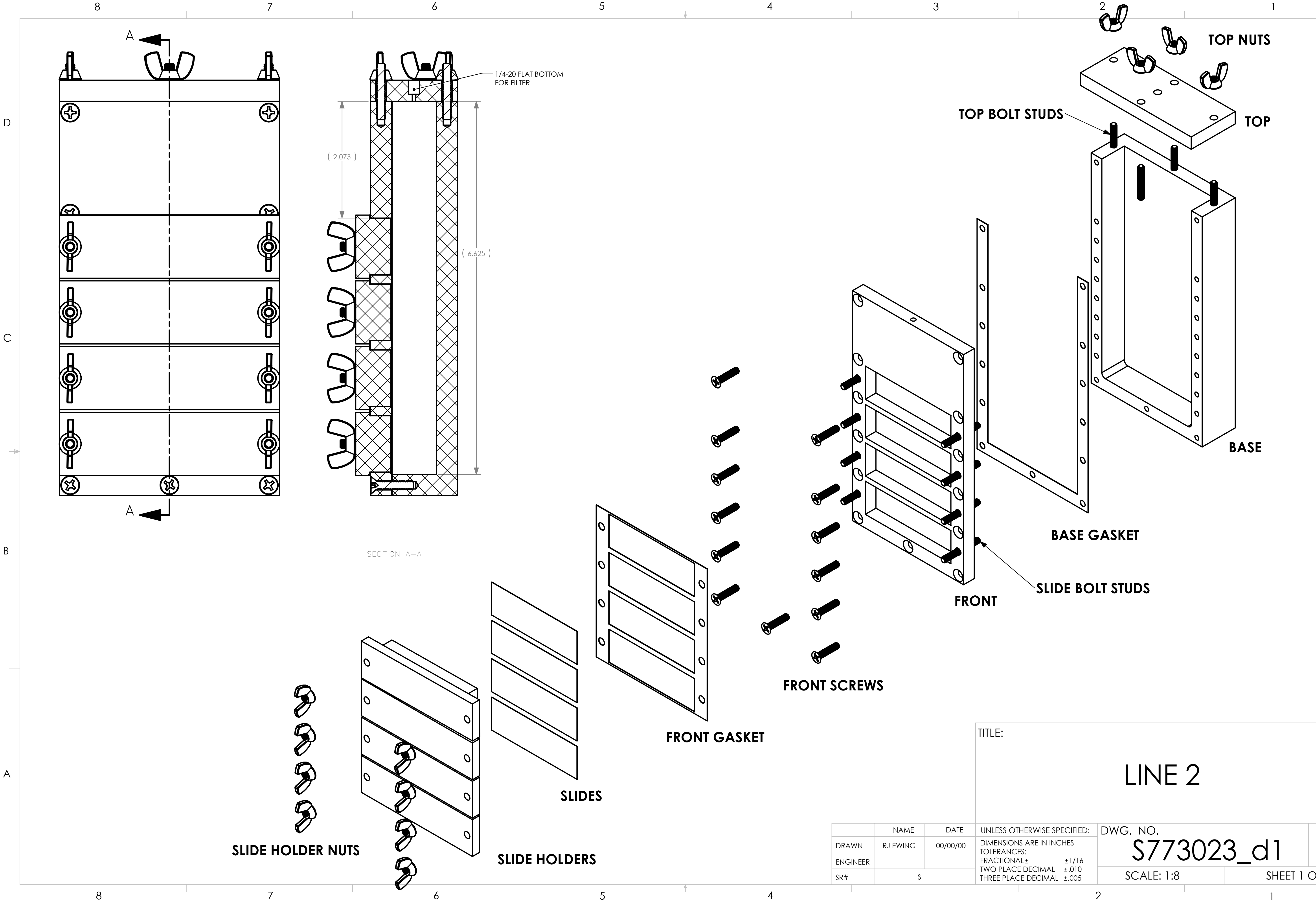

TITLE:

LINE 2

	NAME	DATE	UNLESS OTHERWISE SPECIFIED:	DWG. NO.	REV
DRAWN	RJ EWING	00/00/00	DIMENSIONS ARE IN INCHES<br>TOLERANCES:<br>FRACTIONAL ± ±1/16<br>TWO PLACE DECIMAL ±.010<br>THREE PLACE DECIMAL ±.005	S773023_d1	0
ENGINEER					
SR#	S			SCALE: 1:8	SHEET 1 OF 1
