# Supplementary material for: Visualizing Microbial Community Dynamics via a Controllable Soil Environment
Source: mSystems. 2020 Feb 11;5(1):e00645-19. doi: 10.1128/mSystems.00645-19 (PMC7018529; doi:10.1128/mSystems.00645-19)
Supplement: TEXT S1 [file mSystems.00645-19-s0001.docx]

**Supplementary Information for:**

**Visualizing microbial community dynamics via a controllable soil environment**

Arunima Bhattacharjee^1^, Dusan Velickovic^1^, Thomas W. Wietsma^1^, Sheryl L. Bell^1^, Janet K. Jansson^2^, Kirsten S. Hofmockel^1,3^, Christopher R. Anderton^1*^

^1^Environmental Molecular Sciences Division; ^2^Biological Sciences Division, Earth and Biological Sciences Directorate, Pacific Northwest National Laboratory, Richland, WA; ^3^Department of Ecology, Evolution and Organismal Biology, Iowa State University, Ames IA, 50010

[*Christopher.Anderton@pnnl.gov](mailto:*Christopher.Anderton@pnnl.gov); 902 Battelle Boulevard, Richland, Washington 99354; 509-371-7970

**Table of contents:**

**Supplementary methods…………………………………………………………………………2**

MALDI-MSI sample preparation and data acquisition for lipid standards analysis and negative ion mode imaging**………………………………………………………………………..2**

**Supplementary Methods**

**MALDI-MSI sample preparation and data acquisition for lipid standards analysis and negative ion mode imaging.**

To test potential ion suppression of lipids on chitin islands, a MALDI target plate (MTP 384 Bruker) was rinsed with MeOH and wiped dry using a KimWipe (Kimberly-Clark). Then 1 µl aliquots of the 0.4% colloidal chitin solution was drop-casted and dried, which formed a thin film of chitin on the plate. After which, 0.5 µl aliquots of each PC 34:1, PC 34:0, and PG 34:0 lipid standards (1 µg/µl; Avanti Polar Lipids) were spotted both on- and off-the chitin film (off-film as the control area). As the lipid standard solutions were drying, 1.5 µl of DHB matrix solution (40 mg/ml, 50% aqueous MeOH) was mixed with the lipid mix spot. MALDI-FTICR-MS data was acquired using 200 shots per pixel at 2 kHz frequency, where the mass spectra was averaged over 10 scans on a target spot. Mass spectra was collected over *m/z* 253-2000 in positive ion mode using a 0.57 s transient, which resulted in a mass resolution (R) ~ 170000 at 400 *m/z*. The ions *m/z* 760.5862, 762.6015, and 773.5315 were detected for PC 34:1 [M+H]^+^, PC 34:0 [M+H]^+^, and PG 34:0 [M+Na]^+^, respectively.

To test an alternative approach for visualizing lipids more readily ionizable in negative ion mode, slides retrieved from the SoilBox were prepared using 1, 5-diaminonapthalene **(**DAN) as a MALDI matrix. Here, 10 mg/ml of DAN (70% ACN with 0.2% formic acid) was sprayed using the TM-Sprayer with 4 passes at 50 µl/min, 80 °C, a spray spacing of 2 mm, and a spray velocity of 1200 mm/min. MSI was performed as described in the Methods section of the main text. For MALDI-MSI data acquisition in negative ion mode, the FTICR-MS was operated to collect *m/z* 400-2000 using 50 shots/pixel (at 2 kHz) and a 0.9 s transient (R ~ 260000 at 400 *m/z*).
